# Male–female concordance in reported involvement of women in contraceptive decision-making and its association with modern contraceptive use among couples in rural Maharashtra, India

**DOI:** 10.1186/s12978-021-01187-8

**Published:** 2021-06-30

**Authors:** Anvita Dixit, Nicole E. Johns, Mohan Ghule, Madhusudana Battala, Shahina Begum, Jennifer Yore, Niranjan Saggurti, Jay G. Silverman, Elizabeth Reed, Tarik Benmarhnia, Sarah Averbach, Anita Raj

**Affiliations:** 1 Center on Gender Equity and Health, Division of Infectious Diseases and Global Public Health, School of Medicine, University of California San Diego, La Jolla, USA; 2 Joint Doctoral Program in Public Health (Global Health Track), University of California San Diego/San Diego State University, San Diego, USA; 3 Population Council, New Delhi, India; 4 ICMR-National Institute for Research in Reproductive Health, Mumbai, India; 5 Division of Health Promotion and Behavior, San Diego State University, San Diego, USA; 6 Department of Family Medicine and Public Health, University of California, San Diego, USA; 7 Scripps Institution of Oceanography, University of California, San Diego, USA; 8 Department of Obstetrics, Gynecology, and Reproductive Sciences, School of Medicine, University of California San Diego, La Jolla, USA; 9 Department of Education Studies, Division of Social Sciences, University of California San Diego, La Jolla, USA

**Keywords:** Contraceptive decision-making, Couple concordance, Contraceptive use, Dyadic data, India

## Abstract

**Objective:**

Women’s involvement in contraceptive decision-making increases contraceptive use and reduces unmet need, but study of this has been limited to women’s self-reports. Less research is available examining couple concordance and women’s involvement in contraceptive decision-making as reported by both men and women.

**Study design:**

We carried out a cross-sectional study using data from rural India (N = 961 young married couples). Using multivariable regression we examined the association between concordance or discordance in spousal reports of wife’s involvement in contraceptive decision-making and modern contraceptive use, adjusting for demographics, intimate partner violence, and contraceptive use discussion.

**Results:**

More than one third (38.3%) of women reported current modern contraceptive use. Report of women’s involvement in contraceptive decision-making showed 70.3% of couples agreed that women were involved, jointly or alone (categorized as Concordant 1), 4.2% agreed women were not involved (categorized at Concordant 2), 13.2% had women report involvement but men report women were uninvolved (categorized as Discordant 1), and 12.2% had women report uninvolvement but men report that women were involved (categorized as Discordant 2). Discordant 2 couples had lower odds of modern contraceptive use relative to Concordant 1 couples (adjusted RR = 0.61, 95% CI 0.45–0.83). No other significant differences between Concordant 1 couples and other categories were observed.

**Conclusion:**

One in four couples indicated discordance on women’s involvement in contraceptive decision making, with Discordant 2 category having lower odds of contraceptive use. Couples’ concordance in women’s involvement in contraceptive decision-making offers a target for family planning research and interventions to better meet their needs.

*Trial registration* ClinicalTrial.gov, NCT03514914. https://clinicaltrials.gov/ct2/show/NCT03514914

**Supplementary Information:**

The online version contains supplementary material available at 10.1186/s12978-021-01187-8.

## Plain language summary

Evidence on women’s involvement in decision-making are limited to women’s self reports and often not specific to contraceptive decision-making. This study uses couples dyadic data to assess male–female concordance on women’s involvement in contraceptive decision-making and contraceptive use outcomes. Couple’s concordance on women’s involvement in contraceptive decision-making is associated with contraceptive use. There is potential in couple-focused family planning counseling that enhances women’s contraceptive decision-making agency to improve women’s contraceptive use.

**Supplementary Information:**

The online version contains supplementary material available at 10.1186/s12978-021-01187-8.

## Background

India is home to 20% of the world’s married couples with unmet need for contraceptives, with an estimated 50% of all pregnancies being unintended [1, 2]. Contraceptive use can prevent unintended pregnancy and reduce maternal and child morbidity and mortality [3–5]. Some evidence suggests that women’s control over reproductive decision-making is associated with increased likelihood of contraceptive use in India, though there have been mixed results across studies and other nations [6–10]. This may be a result of women’s control being assessed using women’s self-report only, but couples’ contraceptive decision-making can be better understood by assessing both women and their husbands’ reports. Growing evidence suggests that when couples agree on women’s involvement in decision-making generally, the wife’s healthcare utilization is increased compared to when they disagree [11]. Studies of couples’ dyadic data suggest that balance of power between male and female partners in a couple may improve shared decision-making practices than women’s individual decision-making agency alone [9, 12–16]. However, little research exists examining contraceptive decision-making agency as measured by dyadic couples’ reports.

Men are often the decision-makers for fertility-related issues in India, including contraceptive use [17–20]. Interventions aimed at engaging men in couples’ reproductive health care have been shown to improve contraceptive uptake [19, 21]. Wife’s communication with husband and husbands support of contraceptive use are both associated with improved joint family planning decision-making in these studies [19, 21–23]. However, interventions designed to engage men in contraceptive decision-making have primarily focused on increasing male involvement in family planning, but have not directly addressed women’s perceived decision-making agency. Examination of women’s perceived decision-making agency, through their voice or involvement in the contraceptive decision-making process with their husbands, is warranted.

In this paper, we assess the association between women’s perceived decision-making agency, as measured by couple’s concordance of reporting women’s involvement in contraceptive decision-making, and modern contraceptive use among women in rural India. We also consider the role of intent to use contraception, given the theoretical importance of behavioral intention to perform the outcome [24], and the fact that married women of reproductive age who want to avoid pregnancy and would intend to use contraception still report non-use of contraceptive methods [25, 26]. We also explore the relationship between women’s decision-making agency and women-led contraceptive use, by assessing the association of women’s involvement in contraceptive decision-making with type of contraceptive method used.

## Methods

### Sample

We conducted a cross-sectional analysis using baseline dyadic data collected between September 2018 and June 2019 from the CHARM2 [Counseling Husbands and wives to Achieve Reproductive Health and Marital equity] intervention study of young women (18 to 29 years old) and their husbands in rural Junnar district, Maharashtra, India (N = 1201). Gender matched interviews were carried out in-person separately with husbands and wives using electronic tablets lasting for about 40 minutes. CHARM2 is a two-arm cluster randomized controlled trial (RCT) to evaluate a gender-synchronized, gender-transformative family planning intervention. CHARM2 aims to increase uptake of contraceptives, prevent unintended pregnancy, and decrease interpersonal violence. Couples who were not currently married or cohabiting, or who were using a permanent contraceptive method, were not eligible to participate in the study, in order to meaningfully measure study outcomes, including contraceptive use and unintended pregnancy at follow up. The detailed protocol for this cluster-RCT is published elsewhere [27]. Participants were recruited from households in each of the 20 geographic clusters, with each cluster based on its attachment to a single public sub-health centre catchment area. We then randomized to intervention or control condition at the cluster level; all clusters were identified and randomized prior to study recruitment. The analytic dataset for the current study excluded couples with currently pregnant wives (n = 199) and missing information on decision-making (n = 36). Additionally, one couple missing demographic information and couples using uncommon methods (injectable contraceptive (n = 3) and emergency contraceptive pill (n = 1) were excluded, for a final sample of 961 couples. The University of California San Diego, ICMR-National Institute for Research in Reproductive Health in India, and the Population Council obtained approval from their respective IRBs for the protocol.

### Measures

The primary outcome of interest was women’s report of any current modern contraceptive method (dichotomized as yes/no) based on past three months use. Modern contraceptive methods included were oral contraceptive pills, Intrauterine Devices (IUDs), and male condoms [28]. Our survey included all types of contraceptives that were available in the study area as response options, though only condoms, pills, IUDs, and emergency contraceptive pill are modern spacing methods covered under the public health system. For assessing the association between couples' concordance on women’s contraceptive decision-making agency and women led contraceptive use, methods included were non-modern (withdrawal and rhythm), male condoms, pills and IUDs, where use of pills and IUDs can be considered as women led.

The primary exposure of interest was couples’ perceived women’s contraceptive decision-making agency, and included both wife and husband’s report of wife’s involvement in contraceptive decision-making. Both members were asked, “Would you say that using or not using contraception is: mainly your decision, your husband’s/wife’s, joint by both husband and wife, your mother, mother in law, elderly head of household, your sibling, your husband’s/wife’s sibling or someone else?” The responses were collapsed into four categories of decision-making including woman alone, husband alone, wife and husband jointly, or others. The final couples’ concordance/discordance on women’s involvement in contraceptive decision-making variable was constructed combining husband and wife reports into four categories of contraceptive decision-making:

- Concordant 1 (women and men in agreement): Both agree women were involved (women only or joint decision-making)
- Concordant 2: Both agree that women were uninvolved (men only or others decided).
- Discordant 1: Women report women were involved and men report women were uninvolved
- Discordant 2: Women report women were uninvolved and men report women were involved

Additional variables included a priori as confounders based on previous literature and author expertise were: wife’s age, wife’s education (none or primary, secondary or higher), husband’s age, husband’s education (none or primary, secondary or higher), caste (General, Scheduled Caste/Scheduled Tribe/Other Backward Castes), religion (Hindu, non-Hindu), parity (0, 1, 2–4), any living sons (Yes, No), fertility desires (Have a/another child, No more/none, Undecided/ Don’t know), Below Poverty Line card holder (Yes, No), and wife’s age at marriage. In addition, we included women’s report of ever experience of intimate partner violence (physical and/or sexual), wife’s knowledge of contraceptive methods (number of methods), husbands knowledge of contraceptive methods, and couple concordance of contraceptive discussion in the past 3 months (both yes, both no, Wife yes/Husband no, Wife no/Husband yes). For assessing intention to use, women and men were asked: “Will you use a contraceptive method or continue to use one in the next 3 months to avoid or delay pregnancy?” with a yes/no response.

### Analysis

Descriptive frequencies and proportions were calculated. Multivariable Poisson regression was used to model the relationship between women’s involvement in contraceptive decision-making (reference group: Concordant 1) with modern contraception use for all women, in both an unadjusted and adjusted model for all potential confounders listed above. A Poisson regression with robust variance estimation for confidence intervals was carried out to limit possible inflation in the effect size relative to logistic regression, since the outcome is not rare (modern contraceptive use is greater than 10% in this sample) [29, 30]. All comparison contrasts (comparing Discordant 2 with Concordant 2, Discordant 2 to Concordant 1, and Concordant 1 to Concordant 2) in both unadjusted and adjusted models are reported in Additional file 1: Table S2.

An exploratory analysis to examine contraceptive use intention was carried out with the multivariable model, adjusting for women’s intention to use modern contraceptives (Additional file 1: Table S3 M2), and then men’s intention to use modern contraceptives (Additional file 1: Table S3 M3). Further, an equivalent multinomial logistic regression was carried out with categorical type of contraceptive use as the outcome.

As a sensitivity analysis (Additional file 1: Table S1), a propensity score adjusted Poisson regression was carried out to limit possible selection bias from the observational design of the study. All analyses were conducted using STATA version 14.0 [31].

## Results

A reported 38.3% of wives were using modern contraception: 25.7% were using male condoms, 3.2% pills and 9.1% IUDs. In 70.3% of couples both husband and wife reported that the wife is involved in contraceptive decision-making (Concordant 1) and in 4.2% of couples both husband and wife report that the wife is uninvolved (Concordant 2). Discordance in wife involvement in decision-making was reported by 25.4% of couples, with 13.2% of husbands reporting their wife is uninvolved, while the wife reports she is involved (Discordant 1), and 12.2% of husbands reporting that the wife is involved while the wife reports she is uninvolved (Discordant 2) (Table 1).

**Table 1:** Sociodemographic characteristics of married couples enrolled in CHARM2 in rural Maharashtra, India (N = 961)

Variable	Overall, n (%)	Current modern FP use	
		Yes, n (%)	No, n (%)
Modern contraceptive use (3 mo)			
Yes	368 (38.29%)	–	–
No	593 (61.71%)	–	–
Type of contraceptive used (3 mo) (N = 958)			
None	372 (38.83%)	–	–
Withdrawal or rythm	222 (23.17%)	–	–
Male condoms	246 (25.68%)	–	–
Pills	31 (3.24%)	–	–
IUDs	87 (9.08%)	–	–
Couple concordance on contraceptive decision-making			
Concordant 1 (women and men agreement): Women-Involved (women only or joint)	676 (70.34%)	285 (77.45%)	391 (65.94%)
Concordant 2: Women Uninvolved (men only or other)	40 (4.16%)	11 (2.99%)	29 (4.89%)
Discordant 1: Women-Report Women Involved and Men-Report Women Uninvolved	127 (13.22%)	46 (12.50%)	81 (13.66%)
Discordant 2: Women-Report Women Uninvolved and Men-Report Women Involved	1198(12.22%)	26 (7.07%)	92 (15.51%)
Age in years (Mean, SD)	24.11 (2.92)	24.58 (2.85)	23.83 (2.94)
Age at marriage in years (Mean, SD)	19.42 (2.36)	19.49 (2.33)	19.38 (2.39)
Husband’s age in years (Mean, SD)	29.65 (3.70)	30.12 (3.72)	29.35 (3.66)
Education			
No education + Primary	138 (14.36%)	45 (12.23%)	93 (15.68%)
Secondary or higher	823 (85.64%)	323 (87.77%)	500 (84.32%)
Husband’s education			
No education + Primary	134 (13.94%)	44 (11.96%)	90 (15.18%)
Secondary or higher	827 (86.06%)	324 (88.04%)	503 (84.82%)
Religion			
Hindu	893 (92.92%)	336 (91.30%)	557 (93.93%)
Other*	68 (7.08%)	32 (8.70%)	36 (6.07%)
Caste			
General	652 (67.85%)	261 (71.92%)	391 (65.94%)
SC/ST/OBC**	309 (32.15%)	107 (29.08%)	202 (34.06%)
Below Poverty Line (BPL) card holder			
Yes	240 (24.97%)	86 (23.37%)	154 (25.97%)
No	721 (75.03%)	282 (76.63%)	439 (74.03%)
Parity			
0	104 (10.82%)	13 (3.53%)	91 (15.35%)
1	534 (55.57%)	214 (58.15%)	320 (53.96%)
2–5	323 (33.61%)	141 (38.32%)	182 (30.69%)
Any living sons			
Yes	492 (51.20%)	208 (56.52%)	284 (47.89%)
No	469 (48.80%)	160 (43.48%)	309 (52.11%)
Fertility desires			
Have a/another child	573 (59.63%)	200 (54.35%)	373 (62.90%)
No more/none	314 (32.67%)	135 (36.68%)	179 (30.19%)
Undecided/Don’t know	74 (7.70%)	33 (8.97%)	41 (6.91%)
Knowledge of contraceptive methods (Mean, Range)	4.19 (0–12)	4.50	4.00
Husband’s knowledge of contraceptive methods (Mean, Range)	4.12 (0–11)	4.20	4.07
IPV (Physical or Sexual)***			
Yes	109 (11.34%)	34 (9.24%)	75 (12.65%)
No	852 (88.66%)	334 (90.76%)	518 (87.35%)
Couple concordance on contraceptive discussion			
Both yes	247 (25.70%)	155 (42.12%)	92 (15.51%)
Both no	261 (27.16%)	42 (11.41%)	219 (36.93%)
Wife yes/Husband no	111 (11.55%)	56 (15.22%)	55 (9.27%)
Wife no/Husband yes	342 (35.59%)	115 (31.25%)	227 (38.28%)
Intention to use modern contraceptive in 3mo			
Yes	484 (50.36%)	349 (94.84%)	135 (22.77%)
No	477 (49.64%)	19 (5.16%)	458 (77.23%)
Total N	**961** (100%)	**368** (100%)	**593** (100%)

Excluded 240 women from 1,201 who were either pregnant (199), missing on decision-making responses (37), used an uncommon contraceptive (4), or were missing on a demographic variable (1). Mean (SD/range) are reported for continuous variables. Proportions are reported for categorical variables

^*^Other religion includes Muslim/Buddhist/Jain/Christian/Other

^**^SC: Scheduled Caste, ST: Scheduled Tribe, OBC: Other Backward Caste

^***^IPV includes report of any physical or sexual intimate partner violence, not emotional violence

Adjusted multivariable analysis showed that couples in the Discordant 2 category for contraceptive decision-making (women report women were uninvolved and men report women were involved), had lower odds of reported modern contraceptive use relative to Concordant 1 (women and men agree that women were involved) couples (adjusted RR = 0.61, 95% CI 0.45–0.83), after adjusting for confounders (Table 2). None of the remaining categories of couple concordance on women’s involvement in contraceptive decision-making were significantly associated with the outcome. Exploratorily, we also adjusted for women’s intention to use modern contraceptives, and found that the association of Discordant 2 category for contraceptive decision making with modern contraceptive use relative to Concordant 1 couples was lost. However, once we adjusted for men’s intention to use modern contraceptives, couples in the Discordant 2 category for contraceptive decision-making (women report women were uninvolved and men report women were involved) had lower odds of modern contraceptive use relative to Concordant 1 couples (Additional file 1: Table S3 M3: adjusted RR = 0.61, 95% CI 0.45–0.83), findings comparable to our main findings on Discordant 2 couples. The sensitivity analysis showed that the Poisson adjusted regression with propensity scores did not substantially differ from the adjusted Poisson regression findings, computing a similar magnitude estimate as seen in our main findings (adjusted RR = 0.51, 95% CI = 0.36–0.73) (Additional file 1: Table S2).

**Table 2:** Unadjusted and adjusted poisson regression between couple concordance of women’s involvement in contraceptive decision making and current modern contraceptive use among married couples enrolled in CHARM2 in rural Maharashtra, India (N = 961)

Variable	Unadjusted	Adjusted
*Couple concordance of women’s involvement in contraceptive decision making*	RR (95% CI)	RR (95% CI)
Concordant 1 (women and men agreement): Women-Involved (women only or joint)	ref	ref
Concordant 2: Women Uninvolved (men only or other)	0.64 (0.39–1.04)	0.79 (0.54–1.18)
Discordant 1: Women-Report Women Involved and Men-Report Women Uninvolved	0.82 (0.64–1.05)	0.82 (0.66–1.02)
Discordant 2: Women-Report Women Uninvolved and Men-Report Women Involved	0.52 **(0.38–0.72)**	0.61 **(0.45–0.83)**

Adjusted for age, age at marriage, husbands age, education, husband’s education, caste, religion, parity, any living sons, and Below Poverty Line status, knowledge of family planning methods, fertility desires, husband’s knowledge of family planning methods, physical or sexual IPV, and concordance of FP discussion. ORs in bold represent statistically significant difference at 95% confidence interval

In the multinomial logistic regression with type of contraceptive used as the outcome, Discordant 2 couples had lower odds of reporting condom use and IUD use relative to Concordant 1 couples (Condoms: AOR = 0.49, 95% CI 0.26–0.92, and IUD: AOR = 0.37, 95% CI 0.16–0.89), after adjusting for confounders (Table 3). There were no observed relationships between decision-making concordance and non-modern (withdrawal and rhythm) methods or pill use.

**Table 3:** Unadjusted and adjusted multinomial logistic regression between couple concordance of women’s involvement in contraceptive decision making and type of contraceptive use among married couples enrolled in CHARM2 in rural Maharashtra, India (N = 958)

Variable	Unadjusted	Adjusted
	OR (95% CI)	AOR (95% CI)
*Non-modern (withdrawal, rhythm)*		
Concordant 1 (women and men agreement): Women-Involved (women only or joint)	Ref	Ref
Concordant 2: Women Uninvolved (men only or other)	1.32 (0.62–2.83)	1.35 (0.59–3.11)
Discordant 1: Women-Report Women Involved and Men-Report Women Uninvolved	1.01 (0.62–1.65)	0.84 (0.49–1.42)
Discordant 2: Women-Report Women Uninvolved and Men-Report Women Involved	0.75 (0.46–1.22)	0.86 (0.51–1.47)
*Male condoms*		
Concordant 1 (women and men agreement): Women-Involved (women only or joint)	Ref	Ref
Concordant 2: Women Uninvolved (men only or other)	0.75 (0.32–1.74)	0.98 (0.38–2.52)
Discordant 1: Women-Report Women Involved and Men-Report Women Uninvolved	0.96 (0.60–1.54)	0.86 (0.50–1.48)
Discordant 2: Women-Report Women Uninvolved and Men-Report Women Involved	0.40 **(0.23–0.70)**	0.49 (0.26–0.92)
*Pills*		
Concordant 1 (women and men agreement): Women-Involved (women only or joint)	Ref	Ref
Concordant 2: Women Uninvolved (men only or other)	**	**
Discordant 1: Women-Report Women Involved and Men-Report Women Uninvolved	0.52 (0.15–1.78)	0.34 (0.09–1.29)
Discordant 2: Women-Report Women Uninvolved and Men-Report Women Involved	**	**
*IUD*		
Concordant 1 (women and men agreement): Women-Involved (women only or joint)	Ref	Ref
Concordant 2: Women Uninvolved (men only or other)	0.43 (10–1.90)	0.53 (0.11–2.47)
Discordant 1: Women-Report Women Involved and Men-Report Women Uninvolved	0.48 (0.21–1.10)	0.42 (0.18–1.00)
Discordant 2: Women-Report Women Uninvolved and Men-Report Women Involved	0.38 **(1.17–0.87)**	0.37 **(0.16–0.89)**

Adjusted for age, age at marriage, husbands age, education, husband’s education, caste, religion, parity, any living sons, and Below Poverty Line status, knowledge of family planning methods, fertility desires, husband’s knowledge of family planning methods, physical or sexual IPV, and concordance of FP discussion. ORs in bold represent statistically significant difference at 95% confidence interval

Figures in bold indicate that the differences are statistically and significantly different from zero at the 95% confidence level

^**^Empty cell could not be calculated

## Discussion

One in three couples reported that women were either not involved or had discordant views on women’s involvement in contraceptive decision-making (i.e. they reported Concordant 2, Discordant 1 or Discordant 2). This highlights that many women are not involved in contraceptive decision-making, and many couples are not on the same page about women’s involvement in this decision-making. Discordant 2 couples, where women report women were uninvolved and men report women were involved, had lower odds of contraceptive use compared to Concordant 1 couples, where men and women both agree that women were involved in contraceptive decision-making. One in nine women in our sample report no contraceptive decision-making control while their husbands disagree reporting that their wife is involved. This suggests that some spouses may believe the other to be in control of contraceptive decisions when in fact neither is engaged. This also suggests that some women do not know or do not act on their reproductive agency when their husbands indicate they have it. This could reflect several realities, including poor communication, dismpowerment for the women, or abdication of responsibility by the husbands on contraceptive decision-making.

Although, previous studies assessing women’s responses to contraceptive decision-making suggest that increasing women’s reported agency alone may increase contraceptive use [10], couples concordant report of wife-involved decision-making did not show increased contraceptives use in our sample. On the other hand, couples where women report being uninvolved but men report their wives were involved in decision-making had lower odds of contraceptive use. Comparably, a study of couples’ household decision-making and contraceptive use in Bangladesh suggests that a balance in power, rather than wife only decision-making, may have the most impactful outcomes [9]. Furthermore, the association was not explained by socio-demographic correlates, and only a small part of this association is explained by spousal communication about contraceptives. Couple concordance in report of recent contraceptive discussion was significantly associated with increased modern contraceptive use. In India, greater women’s empowerment has previously been reported among couples who are concordant in their reporting of contraceptive communication and use [12]. Thus, couple communication may explain discordance in decision-making further and should be considered in furture research.

Among Discordant 2 couples (women report women uninvolved and men report women involved), when adjusted for women’s intent to use (Additional file 1: Table S3 M2), an association was not noted with women’s use of contraceptives. Whereas, when adjusted for men’s intent to use, women had lower odds to use contraceptives (Additional file 1: Table S3 M3). Thus, contraceptive use intention plays an important role when men and women disagree on women’s involvement in contraceptive decision-making. However, intention is a complex construct, assumed to be a conscious decision, but can be ambivalent and changing over time [32, 33]. When we assessed the association with type of contraceptive used as the outcome, couples had lower odds to report using condoms and IUDs when women reported that they were not involved in decision-making, but men reported that women were involved (Discordant 2). Although, we expected low use of women-controlled methods, it is also critical for male controlled method of condoms and not specific to women-controlled methods. This highlights the need for women to be able to practice their contraceptive decision-making agency in partnership with men regardless of whether the contraceptive is women controlled or not.

The current study extends our understanding of women’s contraceptive-specific agency by assessing both partners’ report of decision-making, and adds to our understanding that increasing women’s decision-making agency should be accompanied by engaging male partners when possible to optimally improve contraceptive utilization. This is highlighted by the association of discordant couples (Discordant 2) with lower modern contraceptive use. It is further emphasized by the importance of men’s intention to use modern contraception rather than women’s intention to use modern contraception. However, our findings should be considered in the context of several limitations. First, this is a cross-sectional analysis which precludes assumptions of causality. Responses were subject to social desirability bias, and some men may have erroneously reported that their wives were involved in contraceptive decision-making. The sample for this study is from participants enrolled in a RCT, and generalizability of the findings may be limited [27]. In particular, since the CHARM2 intervention aims at improving contraceptive use, only non-sterilized couples were included in the study and this sample, thus underestimating true contraceptive prevalence. Given the Indian context where female sterilization dominates (63.9% of all) contraceptive use [26], our findings are relevant to decisions involving the use of short- and long-term reversible contraceptives (IUD, pills, and condoms) only. These are the only modern methods currently available in the public health system in India and the most common methods reported in the study sample. While it was exploratory, a low cell count limited our understanding of Concordant 2 group among those who do not intend to use contraceptives, and Concordant 2 and Discordant 2 groups among pill users in the multinomial analysis. Improved measurement of women’s decision-making involvement is needed to advance our understanding of this complex construct [34]. Finally, although we used the same measures for husbands and wives’, they may perceive and respond to them differently. Multi-national evidence suggests that men and women don’t have the same cognitive or semantic understanding of response categories to survey questions on gender relations (35).

## Conclusion

To conclude, supporting a more equitable balance of power between couples and encouraging couples’ informed and respectful joint decision-making regarding contraceptive use is important, but may not be enough to create impact. Interventions need to focus on a) women’s agency to be involved and be an active agent in contraceptive decision-making combined with b) male responsibility in family planning and their engagement in family planning programs.

## Supplementary Information

**Additional file 1: Table S1:** Sensitivity analysis showing propensity score adjusted poisson regression for the association between couple concordance of women’s involvement in contraceptive decision making and current modern contraceptive use among married couples enrolled in CHARM2 in rural Maharashtra, India (N = 961).**Table S2:** Unadjusted and adjusted poisson regression for all category comparisons of the association between couple concordance of women’s involvement in contraceptive decision making and current modern contraceptive use among married couples enrolled in CHARM2 in rural Maharashtra, India (N = 961).**Table S3:** Adjusted poisson regression between couple concordance of women’s involvement in contraceptive decision making and current modern contraceptive use women’s intention (M2), and men’s intention (M3) to use modern FP in 3 months, among married couples enrolled in CHARM2 in rural Maharashtra, India (N = 961).

## Data Availability

Due to ethical restrictions regarding patient privacy, data are available upon request from geh@ucsd.edu with subject line “request for CHARM2 data”. Data will be made publically available pending primary evaluation study publication after the end of CHARM2 study.

## Abbreviations

AOR: Adjusted odds ratio
BPL: Below poverty line
CHARM2: Counseling husbands and wives to achieve reproductive health and marital equity 2
CI: Confidence interval
IUD: Intrauterine device
IPV: Intimate partner violence
OBC: Other backward caste
OR: Odds ratio
RCT: Randomized controlled trial
RR: Relative risk
SC: Scheduled caste
SD: Standard deviation
ST: Scheduled tribe
