# Supplementary material for: Male–female concordance in reported involvement of women in contraceptive decision-making and its association with modern contraceptive use among couples in rural Maharashtra, India
Source: Reprod Health. 2021 Jun 30;18:139. doi: 10.1186/s12978-021-01187-8 (PMC8244175; doi:10.1186/s12978-021-01187-8)
Supplement: Supplementary file 1 — Additional file 1: Table S1: Sensitivity analysis showing propensity score adjusted poisson regression for the association between couple concordance of women’s involvement in contraceptive decision making and current modern contraceptive use among married couples enrolled in CHARM2 in rural Maharashtra, India (N = 961).Table S2: Unadjusted and adjusted poisson regression for all category comparisons of the association between couple concordance of women’s involvement in contraceptive decision making and current modern contraceptive use among married couples enrolled in CHARM2 in rural Maharashtra, India (N = 961).Table S3: Adjusted poisson regression between couple concordance of women’s involvement in contraceptive decision making and current modern contraceptive use women’s intention (M2), and men’s intention (M3) to use modern FP in 3 months, among married couples enrolled in CHARM2 in rural Maharashtra, India (N = 961). [file 12978_2021_1187_MOESM1_ESM.docx]

**Table S1:** Sensitivity analysis showing propensity score adjusted poisson regression for the association between couple concordance of women’s involvement in contraceptive decision making and current modern contraceptive use among married couples enrolled in CHARM2 in rural Maharashtra, India (N=961).

| Variable |  |
| --- | --- |
| Couple concordance of women’s involvement in contraceptive decision making | RR (95% CI) |
| Concordant 1 (women and men agreement): Women-Involved (women only or joint) | ref |
| Concordant 2: Women Uninvolved (men only or other) | 0.69 (0.42-1.14) |
| Discordant 1: Women-Report Women Involved and Men-Report Women Uninvolved | 0.86 (0.67-1.10) |
| Discordant 2: Women-Report Women Uninvolved and Men-Report Women Involved | **0.51 (0.36-0.73)** |

**Table S2:** Unadjusted and adjusted poisson regression for all category comparisons of the association between couple concordance of women’s involvement in contraceptive decision making and current modern contraceptive use among married couples enrolled in CHARM2 in rural Maharashtra, India (N=961).

| Variable | Unadjusted | Adjusted |
| --- | --- | --- |
| Couple concordance of women’s involvement in contraceptive decision making | RR (95% CI) | RR (95% CI) |
| Ref Concordant 2 |  |  |
| Concordant 1 (women and men agreement): Women-Involved (women only or joint) | 1.56 (0.96-2.54) | 1.26 (0.85-1.86) |
| Concordant 2: Women Uninvolved (men only or other) | ref | ref |
| Discordant 1: Women-Report Women Involved and Men-Report Women Uninvolved | 1.29 (0.81-2.04) | 1.03 (0.69-1.53) |
| Discordant 2: Women-Report Women Uninvolved and Men-Report Women Involved | 0.81 (0.58-1.15) | 0.77 (0.56-1.05) |
| Ref Discordant 1 |  |  |
| Concordant 1 (women and men agreement): Women-Involved (women only or joint) | 1.21 (0.95-1.54) | 1.22 (0.98-1.51) |
| Concordant 2: Women Uninvolved (men only or other) | 0.77 (0.49-1.22) | 0.97 (0.65-1.44) |
| Discordant 1: Women-Report Women Involved and Men-Report Women Uninvolved | ref | ref |
| Discordant 2: Women-Report Women Uninvolved and Men-Report Women Involved | 0.63 (0.48-0.84) | 0.75 (0.56-0.99) |
| Ref Discordant 2 |  |  |
| Concordant 1 (women and men agreement): Women-Involved (women only or joint) | 1.91 (1.38-2.63) | 1.63 (1.21-2.21) |
| Concordant 2: Women Uninvolved (men only or other) | 1.22 (0.86-1.73) | 1.30 (0.95-1.78) |
| Discordant 1: Women-Report Women Involved and Men-Report Women Uninvolved | 1.57 (1.19-2.08) | 1.34 (1.01-1.77) |
| Discordant 2: Women-Report Women Uninvolved and Men-Report Women Involved | ref | ref |

**Table S3:** Adjusted poisson regression between couple concordance of women’s involvement in contraceptive decision making and current modern contraceptive use women’s intention (M2), and men’s intention (M3) to use modern FP in 3 months, among married couples enrolled in CHARM2 in rural Maharashtra, India (N=961).

| Variable | M2: Adjusted (women’s intent FP in 3 m) | M3: Adjusted (men’s intent to use modern FP in 3m |
| --- | --- | --- |
| Couple concordance of women’s involvement in contraceptive decision making | RR (95% CI) | RR (95% CI) |
| Concordant 1 (women and men agreement): Women-Involved (women only or joint) | ref | ref |
| Concordant 2: Women Uninvolved (men only or other) | 0.93 (0.78-1.11) | 0.83 (0.58-1.20) |
| Discordant 1: Women-Report Women Involved and Men-Report Women Uninvolved | 0.85 (0.70-1.03) | 0.93 (0.77-1.13) |
| Discordant 2: Women-Report Women Uninvolved and Men-Report Women Involved | 0.82 (0.62-1.07) | **0.68 (0.51-0.89)** |

*Note:*

M2: Main adjusted model Table 2 + women’s intention to use contraceptives in next 3 months.

M3: Main adjusted model Table 2 + men’s intention to use contraceptives in next 3 months.
